# Simulated MRI Artifacts: Testing Machine Learning Failure Modes

**DOI:** 10.34133/2022/9807590

**Published:** 2022-11-01

**Authors:** Nicholas C. Wang, Douglas C. Noll, Ashok Srinivasan, Johann Gagnon-Bartsch, Michelle M. Kim, Arvind Rao

**Affiliations:** ^1^Department of Computational Medicine and Bioinformatics, University of Michigan, USA; ^2^Department of Biomedical Engineering, University of Michigan, USA; ^3^Department of Radiology, University of Michigan, USA; ^4^Department of Radiology, Division of Neuroradiology, University of Michigan, USA; ^5^Rogel Cancer Center, University of Michigan, USA; ^6^Frankel Cardiovascular Center, University of Michigan, USA; ^7^Department of Statistics, University of Michigan, USA; ^8^Department of Radiation Oncology, University of Michigan, USA

## Abstract

*Objective*. Seven types of MRI artifacts, including acquisition and preprocessing errors, were simulated to test a machine learning brain tumor segmentation model for potential failure modes. *Introduction*. Real-world medical deployments of machine learning algorithms are less common than the number of medical research papers using machine learning. Part of the gap between the performance of models in research and deployment comes from a lack of hard test cases in the data used to train a model. *Methods*. These failure modes were simulated for a pretrained brain tumor segmentation model that utilizes standard MRI and used to evaluate the performance of the model under duress. These simulated MRI artifacts consisted of motion, susceptibility induced signal loss, aliasing, field inhomogeneity, sequence mislabeling, sequence misalignment, and skull stripping failures. *Results*. The artifact with the largest effect was the simplest, sequence mislabeling, though motion, field inhomogeneity, and sequence misalignment also caused significant performance decreases. The model was most susceptible to artifacts affecting the FLAIR (fluid attenuation inversion recovery) sequence. *Conclusion*. Overall, these simulated artifacts could be used to test other brain MRI models, but this approach could be used across medical imaging applications.

## 1. Introduction

Machine learning algorithms have become more prevalent in clinical research and many other applications. However, many of these algorithms, including neural networks, remain largely uninterpretable in their decision-making process and act as black boxes. This is a definite problem in medical research, where the stakes of a prediction are much higher than for tasks such as image tagging for a search engine. As discussed by Cohen et al., one of the known ways machine learning models fail to perform, is because they are not shown difficult conditions or poor quality inputs typical of real clinical practice, [1] creating the foundations for a “data-centric” AI landscape. The goal of this set of experiments is to articulate known failure modes for brain tumor segmentation and create reasonable simulacra to test the effects on these black box models.

Lessons can be taken from other fields, such as aviation or nuclear engineering, where there is a need for consistency and safety in complex systems. One of the tools utilized by engineers to evaluate the safety of systems is Failure Modes and Effects Analysis (FMEA). Potential modes of failure for a system are preemptively discussed and evaluated to build countermeasures and safety protocols. Each potential failure mode is evaluated for its severity, detectability, and occurrence rate, to prioritize the most severe and likely failure modes. This exercise of simulating MRI artifacts and failure modes can be thought of as a test of severity. Many other researchers have built systems for quality control and preprocessing that could ameliorate potential severe failures if a system is found to have a weakness to certain artifacts.

In the space of MRI artifacts, most of the work to date has focused on correction algorithms for improving data quality. There are a wide variety of ways of simulating, then correcting motion artifact in MRI, from physics-based methods to neural network-based algorithms [2–5]. Correction of field inhomogeneity has led to algorithms such as N4ITK to deal with biased intensity, which they simulated with additive Gaussian noise [6]. There are even algorithms to deal with aliased image volumes [7]. Overall, most of these algorithms are focused on providing a tool for improving data quality, whereas these experiments focus on the impact of artifacts on model performance.

A few groups have worked on using simulated failure modes to study their effects on complex models, though generally one type at a time. Eijgelaar et al. show how using sparsified training data can ameliorate issues caused by ordinary data quality variability, such as missing MRI sequences [8]. Nalawade et al. simulated motion artifact using k-space phase alteration and fed this corrupted output into motion correction algorithms. They then used these corrupted and corrected volumes to attempt to predict genetic markers including IDH, MGMT, and 1p/19q codeletion [9]. These other experiments show the importance of understanding how data quality affects model performance in real world applications.

Gliomas, or brain tumors of the glial tissue, were chosen as a testbed for developing simulated artifacts because brain MRIs are relatively standardized and studied. Head MRI studies lack the kinds of breathing artifacts and other scan window inconsistencies that come with abdominal or chest imaging studies. Since MRI is used for other purposes in the brain such as neurodegenerative diseases, a broad array of toolkits and preprocessing pipelines have been developed that can be leveraged. Brain tumor segmentation is also a task that is used as part of machine learning competitions such as MICCAI BRATS, where many teams compete to improve the performance of algorithms to delineate gliomas [10]. Overall, the developed nature of brain tumor segmentation and the clinical urgency of the task make it a good candidate for evaluation beyond simple metrics, using artifacts to evaluate the stability and trustworthiness of models.

Gliomas can have a wide range of pathologies, radiologic appearances, and treatment prognoses. Glioblastoma Multiforme (GBM) is the highest grade of gliomas and have poor prognosis with a median survival of less than 2 years, despite some advances in treatment regimens [11]. The classical imaging presentation of GBMs on MRI includes hyperenhancement in the edematous regions on the FLAIR sequence and hyperenhancement in the solid enhancing portions of the tumor on T1-weighted postcontrast sequence. FLAIR hyperenhancement is thought to be edema or fluid buildup in the regions surrounding the tumor where the tumor is infiltrating into the brain tissue. Enhancement on T1 postcontrast imaging corresponds to blood-brain barrier disruption, as the gadolinium-based contrast agent appears in the tumor tissue [12].

The other broad category of gliomas studied in these experiments was low-grade gliomas (LGG). The categorization of gliomas has evolved somewhat with the rise of genetic markers such as IDH mutation status and 1p/19q codeletion. However, low-grade gliomas can generally be thought of as less aggressive than GBM, though they have the potential to transform into a more aggressive subtype. With the recognition of the importance of genetic markers for subtypes of gliomas, “low-grade glioma” indicating grade I and II gliomas, is becoming less prevalent in the literature because of its lack of specificity [13]. The rise of molecular and genetic markers has made more subtypes differentiable, and some treatments more targeted, so broad categories of tumors are less useful. However, since the data in these experiments is originally from the TCGA-LGG and TCGA-GBM collections, that terminology of LGGs and GBMs will be used throughout this manuscript to indicate the broad differences between these two groups of tumors [14].

On imaging, low-grade gliomas classically tend to be hyperintense in T2 weighted sequences, particularly T2 FLAIR and hypointense on T1-weighted sequences. Unlike GBM, which classically has enhancement on T1-weighted postcontrast sequences, LGGs typically do not show much of this enhancement [15]. They also tend to be more texturally homogeneous compared to GBMs and are often more diffuse looking. That said, they can have more heterogenous imaging presentations across patients because of the wider variety of tumors encompassed under the label’s umbrella.

## 2. Methods

### 2.1. MRI Artifacts Overview

**Figure 1:**
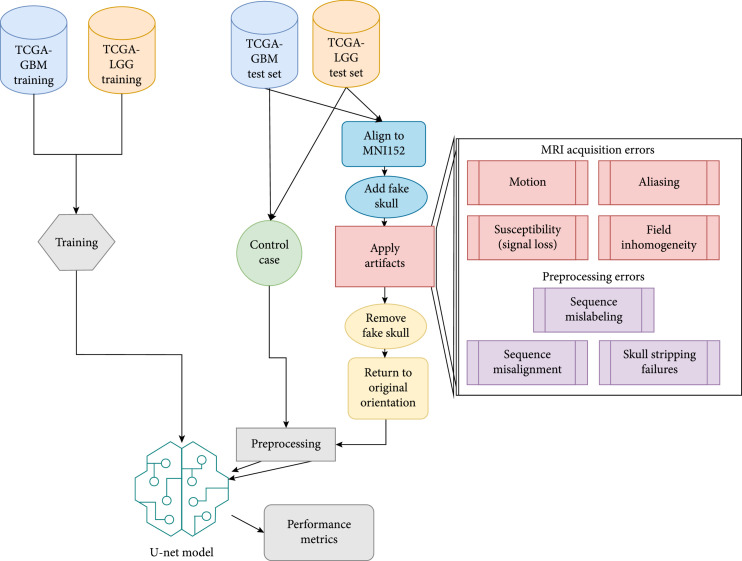
Workflow diagram of the simulated artifacts applied to brain MRI studies. The U-Net model was trained previously on the training data from the MICCAI BRATS 2018 competition, a dataset without simulated artifacts added. The artifacts were evaluated on the held aside testing data from the competition. The seven artifacts simulated were: motion, aliasing, signal loss, field inhomogeneity, sequence mislabeling, sequence misalignment, and skull stripping failures. Scans were aligned to template, had a fake skull added, the artifacts were applied, the fake skull was removed, then the scans were returned to their original orientation. These altered scans were then preprocessed as normal and used as input to the neural network. Metrics were collected to evaluate the performance impact of the simulated artifacts.

A variety of failure modes were used to test the segmentation model across the two types of gliomas. These were developed with the oversight of a board certified neuroradiologist to determine the most common types of artifacts and evaluate the simulated artifacts from a practical perspective. An MRI physics expert was also heavily involved to understand the underlying physics of MRI artifacts and how to sufficiently simulate them. The overarching goal of the experiments is to test the failure modes of models with plausible simulations of those problems. These failure modes included failures during acquisition and preprocessing of these MRI studies. An overview of the workflow in this manuscript is shown in Figure 1. For each experiment, one type of artifact was applied at a time, multiple times across a range of parameters.

### 2.2. Data and Model

#### 2.2.1. Pretrained Model and MICCAI BRATS

The glioblastoma and low-grade glioma brain MRI studies were taken from the TCGA-LGG and TCGA-GBM datasets that were part of the MICCAI BRATS 2018 Challenge [10, 16, 17]. These MRI studies consisted of 4 sequences: T1 weighted precontrast (T1W), T1 weighted postcontrast (T1Gd), T2 weighted imaging (T2W), and T2 Fluid Attenuated Inversion Recovery (FLAIR). Additionally, as these images were part of a segmentation challenge, each had an automated tumor segmentation, with a manually corrected version if necessary. These datasets were each split into a training and testing set as part of the MICCAI Challenge, and this split was used to separate the training and testing sets for the model used in this experiment. A U-Net was trained on the combined GBM and LGG training data to predict the segmentation mask from the four MRI sequences and was previously described in Prabhudesai et al.’s study [18, 19].The training dataset had 103 cases of GBM and 66 cases of LGG, while the test set was split into GBM_test (n=34) and LGG_test (n=44). The goal of this neural network was to predict the tumor mask (combining edema with enhancing and nonenhancing solid tumor) for an MRI study composed of the four standard sequences.

#### 2.2.2. Preprocessing

The MRI volumes were first aligned to the MNI152 template, from Montreal Neurological Institute, to provide a consistency in size and orientation for the studies [20]. The T1W sequence was aligned to the template, then the output transformation was used to transform the other sequences identically. This alignment was performed using a multistage affine registration to sequentially tune the alignment. It starts with a center of mass alignment, followed by translation registration, a rigid registration, then finally an affine registration. These all utilize optimizations from the DIPY software package. The MNI template has isotropic spacing with pixels of size 1 mm×1 mm×1 mm, but after alignment, the original scan sizing may not be precisely the same. Therefore, size parameters are given in pixels in MNI space which are approximately millimeters in original scan space.

#### 2.2.3. Fake Skull Generation

The MRI studies available in the TCGA-GBM and TCGA-LGG datasets were only available in their skull-stripped form for anonymization. However, a rough approximation of the skull was simulated for the studies. This is necessary because of the significance of the skull in various artifacts including motion, aliasing, and when the skull stripping fails.

To simulate the skull, the region 3 to 10 pixels from the boundary of the brain was altered to add a fake skull. This region was changed from black background to a bright region that maxed out at 1.2 times the 80^th^ percentile value of the brain, for a sequence. This skull region has parabolic intensity curve and decreases slightly until it reaches that 3- or 10-pixel distance. This fake skull is clearly not meant to accurately simulate the entire skull, as most of the time the ocular cavity, mandible, and other skull parts do not impact the brain region significantly. However, it is a sufficient approximation for the purposes of this experiment.

### 2.3. Simulated MRI Acquisition Artifacts

**Figure 2:**
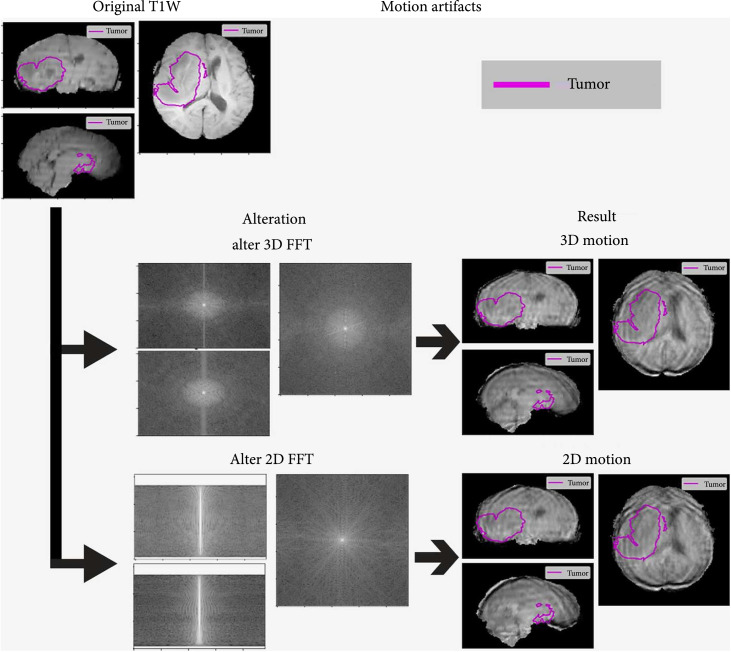
Examples of motion artifacts (3D and 2D). Figures show coronal, sagittal, and axial cuts of the brain MRI for TCGA-14-3477. 3D and 2D FFTs show the image volume after transformation into k-space or the frequency domain. This is where the motion artifacts are applied. The ground truth tumor mask is outlined in blue, to highlight the areas of interest.

**Figure 3:**
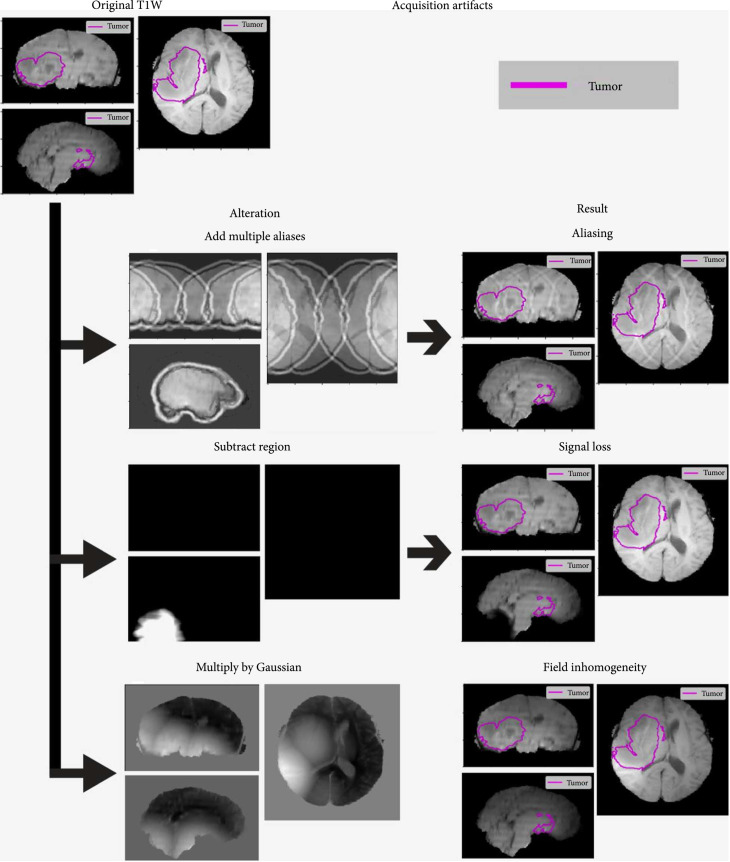
Examples of the other acquisition artifacts: aliasing, signal loss, and field inhomogeneity. Figures show coronal, sagittal, and axial cuts of the brain MRI for TCGA-14-3477. The ground truth tumor mask is outlined in blue, to highlight the areas of interest. The alterations to the original image are shown on the left column, and the resulting altered volumes on the right column.

**Figure 4:**
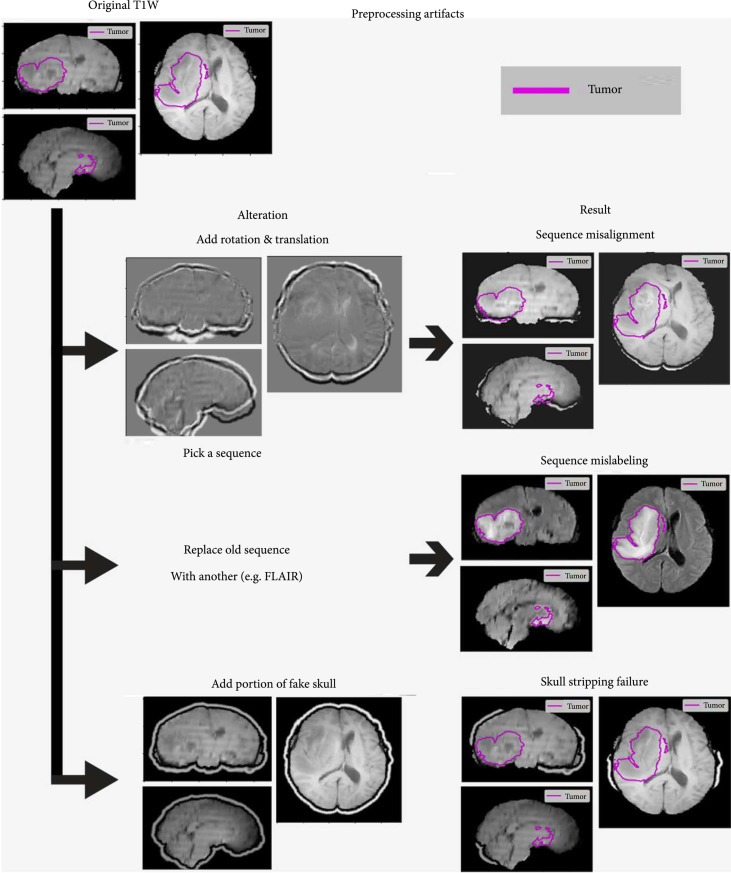
Examples of the preprocessing artifacts: aliasing, signal loss, and field inhomogeneity. Figures show coronal, sagittal, and axial cuts of the brain MRI for TCGA-14-3477. The ground truth tumor mask is outlined in blue, to highlight the areas of interest. The alterations to the original image are shown on the left column, and the resulting altered volumes on the right column.

The MRI acquisition artifacts that were simulated were patient motion, signal loss due to magnetically susceptible materials, aliasing, and field inhomogeneities. Examples of these artifacts can be seen in Figures 2–4. Some of these artifacts were tested in multiple ways, or multiple experiments depending on if they applied to all the sequences or one sequence at a time. Additionally, motion artifacts were simulated using two different methods, one for 3D acquisitions and another for 2D acquisitions. These motion artifacts were also split by the effect of translation vs. rotation and the combined effect of those to movements in a subanalysis. A summary of the generating parameters is included in Table 1, and equations for artifact generation are included in the supplementary materials (available here).

**Table 1:** These are the parameters of the seven types of artifacts simulated independently during the experiments. Only one type of artifact was applied at a time, and the experiments were repeated on the scans across the range of artifact parameters. More specific details on the experiment parameters are included in the methods and supplementary materials (available here).

Artifact type	Split by sequence?	Artifact parameters	Parameter values
Motion	Yes	Translation maximum:	±1 mm to ±12 mm in XYZ
(3D acquisition & 2D acquisition)		Rotation (up-down) maximum:	±3.75° to ±45°
		Rotation (left-right) maximum:	±0.9375° to ±11.25°
Signal loss	No	Artifact base radius:	5 mm to 50 mm
Aliasing	Yes	Number of aliases:	1 to 8 aliases
Field inhomogeneity	No	Maximum intensity increase %:	5% to 40% increase
		Maximum intensity decrease %:	5% to 40% decrease
Sequence mislabeling	Yes	Modality swaps:	One for each combination + no change
Sequence misalignment	Yes	Translation maximum:	±1 mm to ±12 mm in XYZ
		Rotation (up-down) maximum:	±3.75° to ±45°
		Rotation (left-right) maximum:	±0.9375° to ±11.25°
Skull stripping failure	No	Percentage of fake skull:	0%, 1%, 2%, 5%, 10%, 25%, 50%, 100%

#### 2.3.1. Motion Artifacts

Motion artifacts are one of the more common and well described MRI artifacts in the radiology literature. In brain MRI studies, patients can move their heads somewhat during acquisition of the study. As MRI is collected in k-space (frequency domain), movement during acquisition corresponds to different regions of k-space depending on when it occurs and how the scan is acquired. An assumption of a Cartesian acquisition trajectory was used to determine which k-space samples were affected by motion artifact. Additionally, the assumption was made that the phase encoding direction was left-to-right, and the frequency encoding dimension was anterior posterior. These are common settings in brain MRI studies as the short axis of the head is left-to-right [21], however the package that was built for this project can change the encoding directions easily, and other acquisition trajectories could be developed. Both 2D and 3D acquisition modes were simulated by altering how the study was reconstructed after applying an artifact.

The basic scheme of both motion modes is to rotate and translate the volume, then Fourier transform the volume into k-space. In the 3D version, the 3D Fourier Fast Transform (FFT) is applied. For the 2D version, the slices are individually Fourier transformed. This motion and FFT process is performed 10 times to simulate multiple head positions. Then the pixels in k-space are selected that will have motion artifact applied and replaced with the pixels one of the motion-impacted FFTs. The altered k-space is then inverse Fourier transformed back into the spatial domain, and the result is the image volume with a motion artifact applied.

Three different sets of experiments were performed for both the 3D and 2D rotation artifacts. In one case, only rotation was performed, in another only translation, and in the third, both rotation and translation were applied. The rotation consisted of two components, chin up and down (*X*-axis), and head left and right (*Z*-axis). The maximum rotation in the up-down direction was 4 times larger than in the left-right direction because the head is freer to move up and down in a head MRI study. The maximum up and down rotation allowed ranged from 3.75° to 45°, and the left to right rotation was limited between 0.9375° and 11.25°. These rotation magnitudes were chosen from a uniform distribution for each rotated state.

The translation was picked independently for the X, Y, and Z axes, and the shift was randomly selected from a uniform distribution in either the positive or negative directions. The maximum pixel translation was varied in the motion and translation experiments but ranged from 0.5-6 pixels. The full motion artifacts experiment combined both translation and rotation to the original volume.

#### 2.3.2. Susceptibility Artifacts

Susceptibility artifacts due to metallic objects can cause signal loss and distortions in the MRI volume due to rapid variations in the main magnetic field (B0). While the exact effects of a metallic object can be complex and cause false high signals in places, this simulated artifact focused on the darkened signal loss. The size of the signal loss artifact was the main variable under investigation in these experiments. Since the susceptibility artifact is physically present across all sequences, the location of the artifact is the same across all the sequences once set. However, the size of the artifact was 80% smaller on T1W and T1Gd sequences, as an approximation, because T2 weighted sequences have a higher sensitivity to local field changes [22].

The susceptibility artifacts were generated by randomly selecting a seed location outside of the brain, that is also near the tumor. The distance from the tumor was selected to be slightly larger than the size of the signal loss artifact to test local effects on the tumor without directly overlapping the tumor itself. The overall shape of the artifact was spherical, ranging in radius from 5 pixels to 50 pixels. To make the shape more irregular, some smaller spheres were added and subtracted to the map along with some random noise to make the appearance of the artifact more organic. The boundary of the artifact was 10 pixels where signal was dimmed in a gradient rather than set to the background.

#### 2.3.3. Aliasing Artifact

Aliasing occurs in an MRI study when the field of view for the study is smaller than the volume under investigation [23]. The result is that part of the volume wraps around back onto the other side of the volume as a copy in the phase encoding direction. While this aliasing typically is only a single ghosted copy on either side, when employing parallel imaging as a technique, motion artifact can have a similar appearance to multiple aliasing. This aliasing-like artifact with multiple copies was what was simulated in this version. As such, the aliasing artifact was tested across a range of alias copy numbers and was also only applied to one sequence at a time, as this artifact is due to the settings for a single acquisition.

#### 2.3.4. Radiofrequency Field Inhomogeneity

Magnetic field inhomogeneity is when the radiofrequency (B1) magnetic fields are not homogeneous and do not match the values used for calibration. This can cause differences in brightness and darkness in broad and sometimes complicated ways when the reconstruction algorithm fails [24]. There are algorithms for correcting this uneven field such as the N4ITK algorithm and vendor-specific algorithms, that can estimate this bias field and correct it to some extent [6]. These algorithms can also be part of a preprocessing pipeline to help mitigate these inhomogeneities, but it is worthwhile to see what effect they might have on an algorithm’s predictions to begin with.

The overall idea of the inhomogeneity artifact was to create areas of high and low signal which were spread over a large area. The magnetic field inhomogeneity was simulated by randomly selecting 5 seed locations from the area near the edge of the brain. These were used as the high points for the inhomogeneity artifact and were meant to simulate bias in the field near the surface receiver coils. Euclidean distances from these seed locations were used to create spherical Gaussians which were used to multiply the underlying signal with a maximum increase of 1.25× and a maximum decrease of 0.75× by default. The size of the Gaussians was determined by the standard deviation of 75 pixels. The range of maximum increases and decreases was tested from 1.05 to 1.4 and 0.95 to 0.6.

### 2.4. Simulated MRI Preprocessing Artifacts

Some of the artifacts that can emerge in a machine learning pipeline occur during preprocessing of the studies for use by a machine learning algorithm, rather than as part of the physics of acquisition itself. The three artifacts tested in this category included sequence mislabeling, sequence misalignment, and skull stripping failures. Examples of these preprocessing artifacts can also be seen in Figures 2–4. Any practitioner of machine learning algorithms should consider the quality of the data and how well it is handled by a pipeline before signing off on an algorithm. Many of these errors could be caught with quality control and may be possible to remedy even without performing the imaging study again.

#### 2.4.1. MRI Sequence Mislabeling

When deploying an algorithm, it is important to remember that the input data used to train a model is often much cleaner and better organized than real world data. One way this is manifested is the wide variety of MRI sequence names in real world clinical data [25]. MRI study descriptions are named for use by radiologists not machines, and vary by vendor, protocol, and technician, even within a single hospital.

This failure mode was simulated by simply replacing a sequence with a copy of another sequence as though it was mislabeled. An example from an internal dataset, would be if a T1W sequence was mislabeled as FLAIR, because it was called, “Ax T1 Flair”, and the labelling algorithm was only looking the keyword of FLAIR. Contrast can also be tricky to identify by keyword because it might vary from a “+”, or “POST”, or “GAD”, each signifying that contrast had been administered. This simulated artifact is therefore a copied sequence replacing the correct sequence, and then being processed by the algorithm as normal.

#### 2.4.2. MRI Misalignment

Another potential preprocessing artifact is if the MRI sequences are misaligned, due to failure of the sequence alignment on top of one another. Medical image registration is the process of aligning multiple images so that the underlying anatomy is in the same location in the image [26]. While this process is usually performed either during a scan acquisition or early in the preprocessing pipeline, if something did go wrong, it is possible to reregister the scans using a rigid registration algorithm.

This artifact was generated using the same motion method that was used for the motion artifact. However, this time it was applied directly to a single sequence volume rather than through k-space alteration. Before, the motion was applied and then sampled to change the k-space, and motion was performed multiple times, but this motion was applied only once. For comparison’s sake, the amount of motion applied was the same magnitude as the previous motion artifacts.

#### 2.4.3. Skull Stripping Failures

The first of these MRI preprocessing errors to be simulated was the failure of skull stripping, a common preprocessing step in many brain tumor imaging pipelines [27]. Since tumor sometimes has high signal intensity, it can sometimes cause issues for skull stripping algorithms which are looking for similarly high intensity skull pixels to remove. This often manifests in skull stripping algorithms failing to remove skull near the tumor, but also these algorithms can fail to remove skull in other locations. It is possible that these algorithms may confuse tumor with skull and remove that section of the image. However, to give the algorithm the possibility of identifying the tumor, this artifact focused on adding skull rather than subtracting away tumor.

The artifact was simulated by first using the fake skull generation described earlier to add the potential skull but not removing all of it after artifact generation as before. This skull region added is the same across the four sequences because skull stripping is generally performed on the set of sequences to provide a single brain mask. The locations where the skull are added starts near the tumor with randomized locations seeded near the tumor, and then grows as the percentage increases. The percentage corresponds to the amount of fake skull not stripped out over the amount of potential fake skull generated. The percentages of fake skull added in the different experiments were [0%, 1%, 2%, 5%, 10%, 25%, 50%, and 100%].

### 2.5. Artifact Calibration

The artifacts created were first calibrated on an example scan from the TCGA-GBM training dataset. This was to provide a reasonable visual example for the artifacts and to assess the amount of change occurring on these studies. The absolute pixel intensity difference for all the brain pixels was recorded and plotted to show the distribution of change. Additionally representative images of the different artifacts using the default settings on a T1W sequence are shown in Figures 2–4.

### 2.6. Artifact Effect Assessment

The artifacts were assessed on the two different data sets TCGA-LGG and TCGA-GBM and across a wide range of conditions. The primary metrics under investigation was Dice score to see the effect size of each of the artifacts on model predictive power. Other metrics such as area under the receiver operator characteristic (AUROC), sensitivity, specificity, and more were calculated to give a fuller portrait of how the models were affected by these simulated artifacts but are not presented in this manuscript. However, for clarity, the results will focus on Dice score as this is one of the most common metrics used for evaluation of segmentation models.

Additionally, to test for statistical significance, a one-way repeated measures ANOVA test was performed using the statsmodels Python package [28]. The threshold for statistical significance was set at p<0.01 for this project. The Dice scores per patient were compared to the different artifact intensity levels, which were considered “treatments” for the repeated measures ANOVA. These comparisons were performed separately for the LGG and GBM datasets, like the other results. Since the same underlying scans and patients were used in the experiments, this tests the relationship between artifact tuning parameter and Dice score. The artifacts were pseudorandomized by using a seed based on the patient ID, artifact type, and sequence, so as artifact parameters changed, the shape or distribution of an artifact would not vary within a subject. This allow for a more specific inquiry into the effect size of artifacts rather than the variability arising from random generation.

## 3. Results

### 3.1. Baseline Performance of the Model

The model was trained on the combined LGG and GBM training dataset and tested on the held-aside testing datasets from the competition as was previously described in Prabhudesai et al.’s study [19]. The training set had 103 cases of GBM and 66 cases of LGG. The testing set was not used in training or development of the artifacts and was split into two sets: one with 34 GBMs (GBM_test) and one with 44 LGGs (LGG_test). The resulting model had a mean Dice score of 0.907 on the GBM test set. On the LGG test set, the model had a mean Dice score of 0.868. There was also more variance in the Dice scores of the LGG set with a standard deviation of 0.098 as opposed to the GBM’s 0.043. An overview of the mean Dice scores can be seen in Table 2.

**Table 2:** Summary of the mean Dice scores for each of the artifacts at the most extreme artifact levels. Motion artifacts, aliasing, mislabeling, and misalignment were simulated on a per sequence level. Signal loss, inhomogeneous field, and skull stripping failures applied to all the sequences simultaneously.

Artifact	GBM test dataset Dice score at max artifact					LGG test dataset Dice score at max artifact				
	All	FLAIR	T1W	T1Gd	T2W	All	FLAIR	T1W	T1Gd	T2W
Control	0.9073					0.8682				
3D motion		0.7196	0.8641	0.8946	0.8726		0.6029	0.7823	0.8466	0.7974
2D motion		0.7198	0.8641	0.8944	0.8705		0.6054	0.7812	0.8464	0.7978
Signal loss	0.8503					0.7906				
Aliasing		0.8901	0.9024	0.9041	0.9007		0.8150	0.8554	0.8699	0.8645
Inh. Field	0.7684					0.6634				
Mislabeling		0.4920	0.7677	0.8634	0.8579		0.1506	0.6540	0.7766	0.7634
Misalignment		0.6663	0.8798	0.8901	0.8539		0.5227	0.7841	0.8463	0.7940
Skull strip	0.8897					0.8323				

Overall, artifacts on the FLAIR sequence caused the largest decrease in performance for the model, compared to the other sequences. Also, most of the artifacts increased in effect as the artifact was intensified, though this was sometimes subject to nonlinearities such as for the signal loss artifact and diminishing returns as in the skull-stripping artifact. The artifact with the largest impact on model performance was sequence mislabeling, which caused the model to completely miss the tumor on many cases.

### 3.2. MRI Acquisition Artifact Effects

**Figure 5:**
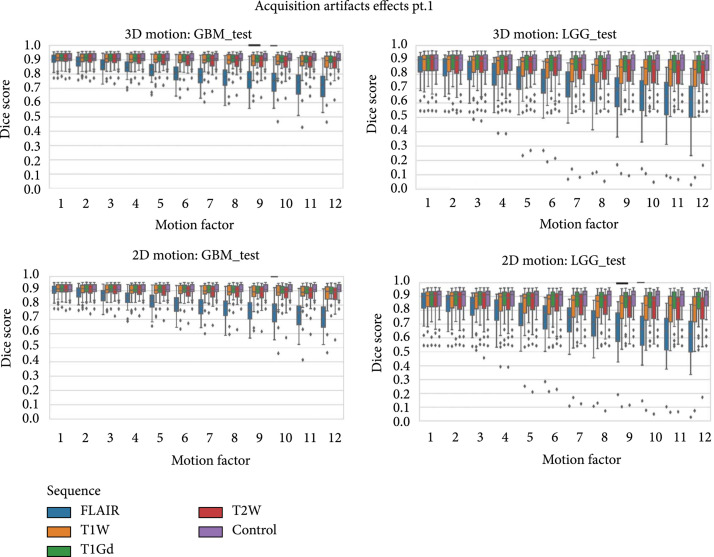
Box plots of Dice scores for the MRI motion artifacts (3D and 2D reconstruction). Motion increases with increased motion factor, and performance tends to decrease with increased motion. Alteration to the FLAIR sequence had the most pronounced effect on model performance.

**Figure 6:**
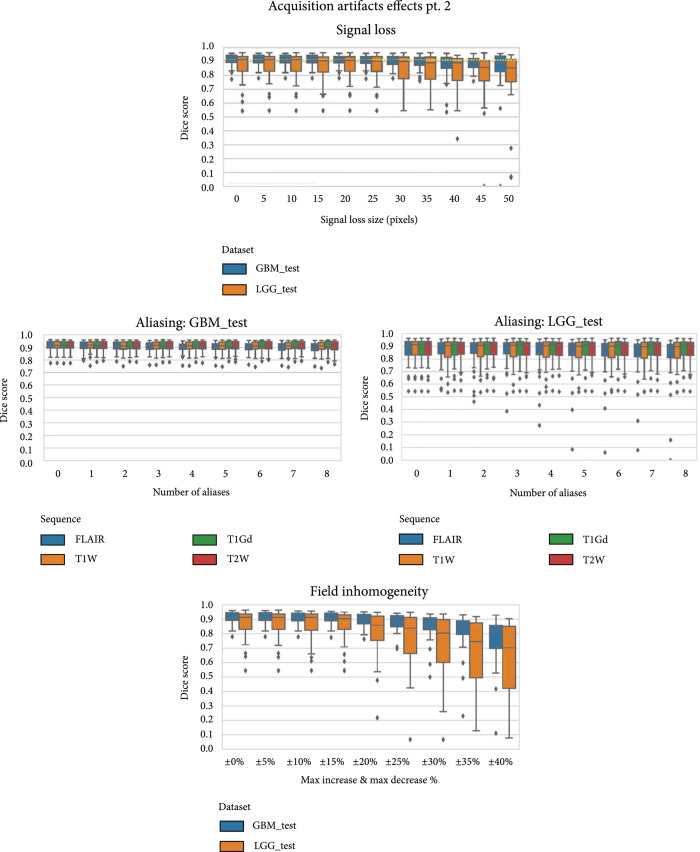
Box plots of Dice scores for the remaining MRI acquisition artifacts: signal loss due to susceptibility, aliasing, and field inhomogeneity. Note that inhomogeneity and signal loss are applied to all the sequences simultaneously, so those subfigures are split by test dataset rather than sequence. Of these artifacts, field inhomogeneity had the largest impact, particularly at high levels of intensity alteration.

The resulting Dice score distributions for these acquisition artifacts across multiple artifact parameters can be seen in Figures 5 and 6. These results are split by test dataset (GBM and LGG), as well as by sequence affected depending on the artifact in question.

#### 3.2.1. Motion Artifact

The 3D motion artifacts showed significant effects for all three version of motion (rotation, translation, both) and had one of the largest effects on performance of the artifacts. For all the motion artifacts though, the largest change was when the artifact was applied to the FLAIR sequence, and the effect of the artifact increased with the increased motion. Though the effects were smaller than the effect of motion on the FLAIR sequence, there were some differences between GBM and LGG cases depending on the sequence affected. For GBM cases, artifacts on T2W imaging and on T1Gd imaging had more effect than on T1W. For LGG cases, there was a stronger association with T2W imaging and T1W imaging than the contrast based T1Gd.

For both LGG and GBM, the effects of translation were smaller than those of rotation, at the maximum amount, though similar. In GBM, at maximum rotation and translation, the Dice score dropped to 0.739 for rotation, 0.758 for translation, and 0.720 for full motion on FLAIR. In LGG, on FLAIR, the Dice score dropped to 0.635 for rotation, 0.656 for translation, and 0.603 for full motion. This pattern held true across the other sequences as well though at a smaller performance drop. Combining rotation and translation into full motion had an additive effect, with a slightly larger drop in model performance.

2D motion artifacts showed a similar pattern where FLAIR was the most affected by motion artifact, for both the GBM and LGG test sets. The results for motion were very similar regardless of whether the reconstruction was performed in 2D or in 3D. For 2D motion, on FLAIR, the Dice score dropped from 0.907 to 0.720 for GBM. On LGG, the drop was from 0.868 to 0.605, which was a marginally smaller drop than 3D motion artifact which dropped to 0.603. Overall, both reconstruction modes of motion resulted in significantly large performance drops when applied to the FLAIR sequence (3D GBM: $p=5.0\text{x}10^{-119}$, 3D LGG: $p=5.6\text{x}10^{-135}$, 2D GBM: $p=5.3\text{x}10^{-119}$, 2D LGG 6.5x10^-139^) and smaller effects on other sequences, with very small differences between the reconstruction modes.

#### 3.2.2. Signal Loss

At small artifact sizes, signal loss outside of the tumor did not cause large changes in model performance [Figure 6]. However, as the signal loss approached 50 pixels in size, starting around 35 pixels or so, the effects of the artifact started to appear and grow in effect. The mean Dice score for the LGG test set decreased from 0.868 to 0.791, with an increased standard deviation of Dice from 0.098 to 0.201. Meanwhile on the GBM test set, the model’s Dice score decreased from 0.907 to 0.850, and the standard deviation of Dice increased from 0.043 to 0.172.

#### 3.2.3. Aliasing

In the aliasing experiments, the number of aliases added to the image generally had an increasing effect on the performance degradation of the model, but this was most pronounced on the FLAIR sequence. For GBM, the change in performance was small but significant ($p=2.5\text{x}10^{-21}$), decreasing from 0.907 to 0.890 with the full 8 aliases on FLAIR. For the LGG test set, the decrease in performance on FLAIR was larger (0.868 to 0.815), but the standard deviation also increased (0.098 to 0.190) causing the statistical linkage to be significant but less so ($p=8.8\text{x}10^{-8}$). Both GBM and LGG showed some decrease in performance when aliasing was performed on T1W imaging, but the other two sequences had much less significant or insignificant changes to performance.

#### 3.2.4. Field Inhomogeneity

Field inhomogeneity had a large and significant effect on the performance of the model, with a more pronounced effect on the TCGA-LGG testing dataset ($p=3.2\text{x}10^{-57}$) than on the TCGA-GBM dataset ($p=6.2\text{x}10^{-27}$). The highest level of artifact was a 40% increase in maximum intensity and a 40% decrease in minimum intensity. For GBM, the mean Dice score, decreased from 0.907 to 0.768, with an increase in Dice standard deviation from 0.043 to 0.167. On the LGG test set, the mean Dice score dropped from 0.878 to 0.663 with a standard deviation increase from 0.098 to 0.222. Overall, field inhomogeneity as an artifact can have a large impact on model performance.

### 3.3. Preprocessing Artifacts Effects

**Figure 7:**
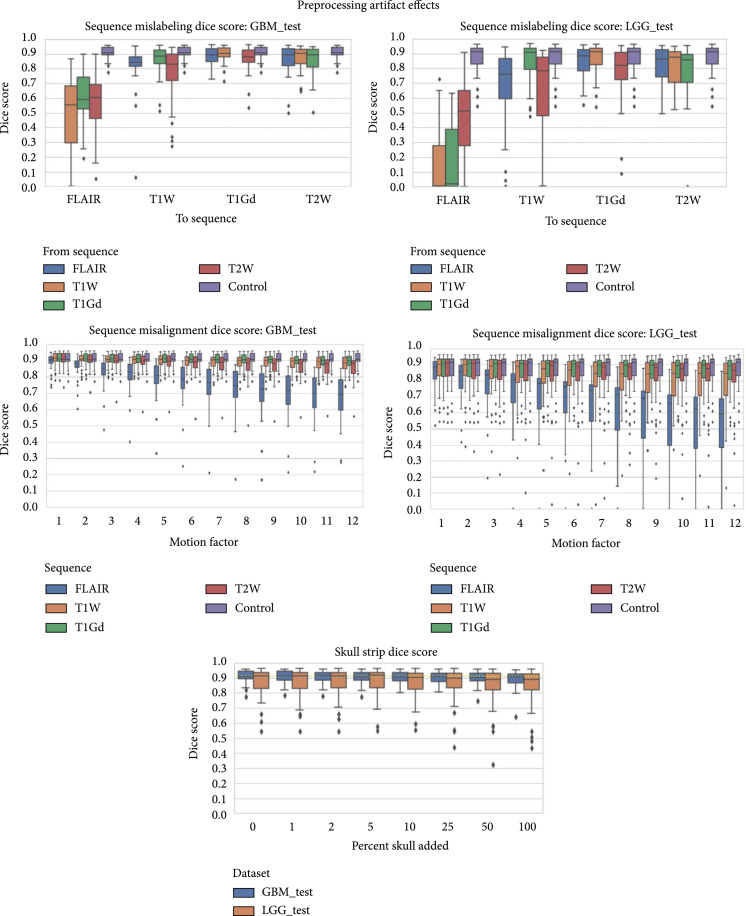
Box plots of Dice scores for the MRI preprocessing artifacts: sequence mislabeling, skull stripping failures, and sequence misalignment. Note that skull stripping failure is applied to all of the sequences simultaneously, so that subfigures are split by test dataset rather than sequence.

The resulting Dice score distributions for these preprocessing artifacts across multiple artifact parameters can be seen in Figure 7. These results are split by test dataset (GBM and LGG), as well as by sequence affected depending on the artifact in question.

#### 3.3.1. Sequence Mislabeling

The largest effect on the Dice score came from mislabeling the sequences, and changes to the FLAIR sequence caused a very large effect. Replacing the FLAIR sequence with one of the T1-based sequences (T1W and T1Gd) made the mean Dice score drop to 0.218 and 0.214, respectively, in the LGG dataset. This effect was similar but less catastrophic for the GBM dataset for which replacing the FLAIR with T1W and T1Gd resulted in Dice scores of 0.492 and 0.607, respectively. Replacing a T1Gd sequence with the T1W sequence had the smallest effect on both LGG and GBM test sets. The reverse of replacing a T1W sequence with T1Gd had a larger impact on performance, decreasing the performance from 0.868 to 0.845 on LGG and 0.907 to 0.848 on GBM.

#### 3.3.2. Sequence Misalignment

Sequence misalignment used the same motion amounts as the motion artifact, but applied the change directly to a sequence, rather than to a portion of k-space. This resulted in a much larger effect on model performance for the same motion than either the 2D or 3D motion artifact. For the FLAIR sequence, this misalignment was also one of the largest changes in performance generated by an artifact, with a drop in Dice score from 0.868 to 0.522 on LGG and 0.907 to 0.666 on GBM. However, it is possible a radiologist would also move their segmentation if they had based it off the FLAIR sequence, but they would likely notice the misalignment.

#### 3.3.3. Skull Stripping

Failures of skull stripping caused increasing performance loss as more of the fake skull was left in place. However, since the progression of skull stripping percentages left in was nonlinear to cover a broader range of values, it is notable there was a slight effect of diminishing returns. For LGG, at 10% of the skull added, the Dice score dropped from 0.8682 to 0.8596 (a 0.0086 drop), at 100% skull added, that dropped to 0.8322 (a 0.0360 drop from baseline). For GBM, the overall effect was smaller. At 10% of the skull added, the Dice score dropped by 0.0027 from 0.9073 to 0.9046 and with 100% skull added, the drop in Dice score was 0.0176 from 0.9073 to 0.8897.

## 4. Discussion

Overall, this set of experiments showed reasonable simulations of known MRI artifacts can be used to test a machine learning model of tumor segmentation and that they can have significant effects. This toolkit was designed to provide segmentation model builders with more tools for testing or perhaps in the future data augmentation. If a clinical team is looking to compare multiple models for automated brain tumor segmentation, they could use these artifacts to understand which models are susceptible to the potential failure modes that crop up in their practice. The exact details of each of the artifacts and their appearance is something that could be debated or altered and hopefully will be built upon by others in the future. However, many competitions and evaluations of models focus only on clean datasets, and small incremental performance increases, without considering real-life problems. For example, the difference in Dice scores between the top 25% of models in MICCAI BRATS 2018 was less than 0.025 [29]. It can be difficult for a machine learning practitioner to evaluate a model beyond the straightforward metrics like Dice score and AUROC without clinical expertise. Likewise, it is difficult for a clinician to translate clinical expertise into something that can be programmed and used as a test case for a model. The combination of these two types of expertise can be used to create more robust models in brain tumor segmentation and many other types of medical imaging applications. This knowledge partnership is also important for prioritizing quality control algorithms for a dataset or clinical implementation, as each artifact will have its own solution, and it always takes additional time to utilize these quality control steps.

This model did not find as large as performance hit to the model when altering sequences other than FLAIR. That FLAIR is important for segmentation seems logical since radiologists often use it to outline the extent of edema. Perhaps this suggests that the model is also using the bright regions of FLAIR to delineate its predictions of the tumor. Then, when FLAIR is affected in ways it has not been trained on, such as motion artifact, its predictive power suffers the most. It is somewhat surprising that T1Gd was not as important to the model’s segmentation as GBM also is characteristically known for enhancement on T1Gd sequences. However, this is likely due to treating the entire tumor as the target rather than attempting to separately segment the different components of the tumor (e.g., edema, solid enhancing tumor, and necrosis). Since edema usually encompasses the solid portion of the tumor, and is bright on FLAIR, that is likely sufficient for the machine learning model to make a prediction without relying heavily on T1Gd. If another model was used that performed the multitarget segmentation that was part of the MICCAI BRATS competition, it would be interesting to see if it was more sensitive to changes on other sequences. An associated interpretation is that, in applications where specific subregions are relevant (like in treatment planning for radiotherapy), this could become a key vulnerability and might need closer examination.

In this experiment, the most straightforward type of failure also had the largest effect on the model. Simply mislabeling the sequence, particularly for the most sensitive sequence (FLAIR), caused the model to be unable to segment the tumor for many cases. While not an artifact in the radiological sense, a traditional machine learning model would not be able to detect that the wrong sequence that had been substituted, and a radiologist would immediately notice that the sequence was not FLAIR. This artifact could easily emerge from differences in the naming of sequences (e.g., T1 FLAIR) and is important to consider in deploying any tumor segmentation model. Proper QC and data engineering procedures can help ensure that this does not happen in a deployed setting. It is notable that the model is not as susceptible for every sequence, so this type of testing can help prioritize detection of mislabeled sequences. Some algorithms, such as work done by Liang et al. have been developed to take DICOM header information to identify sequences with high accuracy [25].

One of the other artifacts that had a very large effect was field inhomogeneity, which caused a performance to drop and the variance of performance to increase. Thankfully, field inhomogeneity is known to be an issue, and many preprocessing pipelines have normalization algorithms to correct for this problem such as N4ITK [6]. The inhomogeneity artifact had a larger effect on the LGG cases than the GBM cases, because LGG cases are generally more subtle than GBM on imaging. The large patches of change might mimic LGG more closely than they do in GBM. Overall, this is a reminder to anyone working on tumor segmentation not to neglect bias field normalization, and this codebase provides a toolset to investigate a model’s susceptibility to field inhomogeneity.

Motion artifact has been well described in the literature, with perhaps the most simulated versions of the artifact of any type from this manuscript [3]. Many papers focus on motion correction algorithms and alter smaller portions of k-space, like k-space lines [2] or only the phase of k-space to simulate artifacts [4]. These methods are reasonable as well but focus on creating correctable artifacts rather than testing to failure. Another potential alteration of the motion artifact generation might use an even-odd slice structure for the 2D reconstruction, which would cause alternating slices with motion [30]. An alternating pattern can even be seen in the sagittal and coronal slices of the example volume. If a model is susceptible to motion artifact and it happens in clinical care, there are plenty of tools for a pipeline to correct this type of artifact.

One of the artifacts that did not produce as much effect on the model was skull stripping. Skull stripping was a source of error when using MICCAI BRATS competition algorithms on an internal dataset but produced limited effects in these experiments. This is likely due to the limited simulation of the fake skull, which may be sufficient for approximating the skull for motion but is quite simple. It would be better to take scans that had the skull intact before skull stripping and use the approach of keeping regions employed by the artifact. However, as these scans came after skull stripping, that is an approach for another project.

While the approach of generating artifacts hopefully can be another axis of testing a model’s robustness, care must be taken before using it as a data augmentation technique. Theoretically these techniques could be used to add artificial artifacts to a training dataset to make a model trained on them potentially more robust. However, the algorithms underlying these simulated artifacts would likely be picked up by a convolutional neural network. That would make the network learn the noise pattern rather than the natural changes induced by artifacts. A cleaner approach would be to include broader range of scan qualities in the training dataset to teach the model about artifacts from real data, using techniques that can handle noisy training data [31]. An interesting future research direction could be to identify known scans with artifacts and compare simulated artifact data augmentation to real world examples.

Additionally, the U-Net is only one type of segmentation model, and many other types and subtypes of machine learning models exist. It may be that models based on classical computer vision techniques, or another convolutional neural network architecture may respond differently to simulated artifacts. As such, other trained models could be used on the simulated artifact image volumes or in other datasets to study their failure modes. Our hope is that this toolbox may eventually be another axis of evaluating segmentation models.

This approach of simulating artifacts can be generalized to other types of medical imaging artifacts and models but requires multidisciplinary cooperation. This approach has been applied to pathology artifacts, by working with pathology literature and pathologists to identify the most likely forms of artifacts and creating simulated versions to apply to tissue slide tiles. Any deployed medical model should be built with clinicians who work in the space and those who knows where things can go wrong. It is also important for data scientists to evaluate their pipelines and think about what quality control steps were needed to turn their original dataset into a clean version the model uses. Existing tools like MRQy or HistoQC can be used for quality control in their respective applications and should be considered for deployed models [32, 33]. We can all work together to build more test cases for our models and improve existing QC tools to create more robust and trustworthy models.

## 5. Conclusion

This manuscript focuses on a new toolkit developed for testing brain tumor MRI models using simulated artifacts. Seven simulated artifacts were developed, four were based on MRI acquisition errors: motion, aliasing, field inhomogeneity, and susceptibility signal loss. The other three were based on pipeline processing failures: sequence mislabeling, sequence misalignment, and skull stripping failure. A convolutional neural network, trained to segment brain tumors, was evaluated on the MICCAI BRATS 2018 dataset. The seven simulated artifacts were applied across a range of artifact strengths, to test the model’s susceptibility to each artifact. Sequence mislabeling, sequence misalignment, motion, and field inhomogeneity caused the largest decrease in model performance, and the model was particularly susceptible to changes on the FLAIR sequence.

## Data Availability

Python code, models, and Jupyter notebooks are available at https://github.com/Systems-Imaging-Bioinformatics-Lab/mri-artifacts. The source brain MRI studies can be found at The Cancer Imaging Archive [14, 16, 17], as part of the MICCAI BRATS 2018 competition. Additional computed metrics beyond Dice score, output files, and image volumes with simulated artifacts are available by request to the corresponding author.

## Supplementary Materials

Supplementary Materials Acquisition Artifact Generation Details. Preprocessing Artifact Generation Details.
