# Supplementary material for: Simulated MRI Artifacts: Testing Machine Learning Failure Modes
Source: BME Front. 2022 Nov 1;2022:9807590. doi: 10.34133/2022/9807590 (PMC10521705; doi:10.34133/2022/9807590)
Supplement: Supplementary Materials — Acquisition Artifact Generation Details. Preprocessing Artifact Generation Details. [file 9807590.f1.docx]

**Supplementary Materials**

1. Acquisition Artifact Generation Details

1.1 Motion Artifact Pseudocode

1. Fourier fast transform image volume

$F\left\{ g\left( x \right) \right\}=\int_{-\infty}^{\infty} g\left( x \right)e^{-i\pi xs}dx=\mathcal{F}\{x\}$

2. Create 10 copies of image volume, and rotate and translate the copies
   $a_{mot}=motion factor [1,12]$
   1. $\theta\sim U\left( -\frac{\pi}{48}a_{mot}, \frac{\pi}{48}a_{mot} \right)$ : left-right rotation
   2. $\varphi\sim U\left( -\frac{\pi}{12}a_{mot},\frac{\pi}{12}a_{mot} \right)$ : up-down rotation
   3. $t_{x,y,z}\sim U\left( -\frac{1}{2}a_{mot},a_{mot} \right)$ : x, y, z translation
3. Fourier fast transform the altered image volumes

$F\{g_{a}\left( x \right)\}=\int_{-\infty}^{\infty} g_{a}\left( x \right)e^{-i\pi xs}dx\mathcal{= F\{}x\}$

4. Replace 10 sections of image volume with altered k-space
   1. Regions are selected to be central using a normal distribution for more consistent/larger effect
   2. $s_{i,j,k} \sim N\left( 0,\frac{1}{4}f_{max} \right)$: where the frequency space ranges from $-f_{max} to f_{max}$
      1. 11 points to define regions, where region n = $s_{n}:s_{n+1}$
   3. Indices are ordered by frequency-encoding dimension, slice dimension, then phase-encoding dimension
5. Inverse Fourier fast transform altered k-space

$F^{-1}\left\{ G_{a}\left( s \right) \right\}= \int_{-\infty}^{\infty} G_{a}\left( s \right)e^{i\pi xs}ds= \mathcal{F}^{-1}\left\{ s \right\}$

6. Replace selected sequence with altered image volume

1.2 Signal Loss Pseudocode

1. Select seed location of signal loss artifact based on tumor and edge of brain $\left( x_{s},y_{s},z_{s} \right)$
   1. Distance from edge of brain = 0:10 pixels
   2. Distance from edge of tumor = ($r_{A}$): ($r_{A}$+ 20 pixels)

$r_{A}=artifact size$

2. Find Euclidean distance from seed location

$D_{A}=\sqrt{\left( x-x_{s} \right)^{2}+\left( y-y_{s} \right)^{2}+\left( z-z_{s} \right)^{2}}$

3. Alter spherical distance with additional passes
   1. Randomly select 100 pts near edge of initial sphere
      $\left( x_{inc},y_{inc},z_{inc} \right) \sim U(r_{a}-7.5\leq D_{A}\leq r_{a}+7.5)$
      $D_{inc}=min(\sqrt{\left( x-x_{inc} \right)^{2}+\left( y-y_{inc} \right)^{2}+\left( z-z_{inc} \right)^{2}}$
      $D_{Ai}= D_{A}-\frac{\left( 10-\max\left( 10,D_{inc} \right) \right)}{2}$
   2. Repeat but inverted
      $\left( x_{dec},y_{dec},z_{dec} \right) \sim U(r_{a}-7.5\leq D_{Ai}\leq r_{a}+7.5)$
      $D_{dec}=min(\sqrt{\left( x-x_{dec} \right)^{2}+\left( y-y_{dec} \right)^{2}+\left( z-z_{dec} \right)^{2}}$
      $D_{Aid}= D_{Ai}+\frac{\left( 10-\max\left( 10,D_{dec} \right) \right)}{2}$
      $D_{F}=filter\left( D_{Aid} \right)$
   3. 2D Median filter to smooth (Kernel size = 15)
4. Create artifact based on distance map

$I_{min}=minimum value of entire image$

$I_{avg}=I_{orig}\left( \frac{D_{F}-R_{A}}{10} \right)+I_{min}(1-\frac{D_{F}-R_{A}}{10})$

$I_{A}=\left\{ \begin{matrix} I_{orig} & D_{F}>R_{A}+10 \\ I_{avg} & R_{A}+10\geq D_{F}\geq R_{A} \\ I_{min} & D_{F}<R_{A} \end{matrix} \right.$

5. Apply to all the sequences using the same seed location
   1. For T1W & T1Gd, $R_{A,T1}=R_{A}*0.8$
   2. Smaller artifact size for the T1 based sequences

1.3 Aliasing Artifact Pseudocode

1. Determine locations of aliased wrap arounds
   1. Evenly space aliases based on number selected [0-8]
2. Add a copy of image volume multiplied by 1.1 onto the image
   1. Image is shifted so it is partially overlapping on each side
3. Repeat for number of aliases desired
4. Replace selected sequence with altered image volume

1.4 Inhomogeneity Artifact Pseudocode

1. Select 5 seed locations of inhomogeneity artifact based on tumor and edge of brain $\left( x_{s},y_{s},z_{s} \right)$
   1. Distance from edge of brain = 0:25 pixels
   2. Distance from edge of tumor = 25:50 pixels
2. Create distance map from 5 seed locations

$D_{A}=\min\left( \sqrt{\left( x-x_{s} \right)^{2}+\left( y-y_{s} \right)^{2}+\left( z-z_{s} \right)^{2}} \right)$

3. Intensity change is based on Gaussian function
   $m_{I}=e^{-\frac{1}{2}\left( \frac{D_{A}}{\sigma} \right)^{2}}$
   1. $\sigma=75$ pixels
4. Adjust intensity change by increase and decrease parameter
   1. $c_{max}$ = max increase [1-1.4]
   2. $c_{min}$ = max decrease [1-0.6]

$m_{A}=\max(c_{max} m_{I},c_{min})$

$largest decrease possible=c_{min}$

5. Apply to all the sequences

$I_{A}=m_{A}I_{orig}$

2. Preprocessing Artifact Generation Details

2.1 Sequence Mislabeling Pseudocode

1. Select a sequence to replace
2. Replace it with another sequence (or don’t replace for control)

2.2 Sequence Misalignment Pseudocode

1. Create a copy of image volume, and rotate and translate the copy
   $a_{mot}=motion factor [1,12]$
   1. $\theta\sim U\left( -\frac{\pi}{48}a_{mot}, \frac{\pi}{48}a_{mot} \right)$ : left-right rotation
   2. $\varphi\sim U\left( -\frac{\pi}{12}a_{mot},\frac{\pi}{12}a_{mot} \right)$ : up-down rotation
   3. $t_{x,y,z}\sim U\left( -\frac{1}{2}a_{mot},a_{mot} \right)$ : x, y, z translation
2. Replace selected sequence with misaligned copy

2.3 Skull Stripping Pseudocode

1. Select 5 seed locations for skull stripping failure based on tumor and edge of brain $\left( x_{s},y_{s},z_{s} \right)$
   1. Distance from edge of brain = 0:10 pixels
   2. Distance from tumor = 10:20 pixels
2. Calculate Euclidean distance to the 5 seed locations

$D_{A}=\min\left( \sqrt{\left( x-x_{s} \right)^{2}+\left( y-y_{s} \right)^{2}+\left( z-z_{s} \right)^{2}} \right)$

3. Order pixels in generated fake skull based on distance to seed locations
4. Select all pixels below percentile of choice
   $p=\{0,1,2,5,10,25,50,100\}$
   1. Add those fake skull pixels to the image volume
5. Apply to all the sequences
